# Case series of sporotrichosis at a teaching hospital in Brazil

**DOI:** 10.1590/0037-8682-0509-2019

**Published:** 2020-05-18

**Authors:** Ana Maria Benvegnú, Lia Natália Diehl Dallazzem, Raíssa Massaia Londero Chemello, André Avelino Costa Beber, Diego Chemello

**Affiliations:** 1 Universidade Federal de Santa Maria, Departamento de Dermatologia, Santa Maria, RS, Brasil.; 2 Universidade Federal de Santa Maria, Departamento de Clínica Médica, Santa Maria, RS, Brasil.

**Keywords:** Cross-Sectional Studies, Mycoses, Sporotrichosis

## Abstract

**INTRODUCTION:**

Sporotrichosis is a subcutaneous fungal infection with a worldwide distribution and higher incidence in tropical and subtropical areas, such as the Brazilian territory, where it has been standing out due to its frequent epidemics. Thus, the present study aimed to evaluate the prevalence of sporotrichosis and profile the affected patients at a university teaching hospital in the central region of Rio Grande do Sul, Brazil.

**METHODS:**

This study was a case series of patients diagnosed with *Sporothrix* spp. from January 2006 to December 2015 by microscopic examination or fungal isolates. Medical records were reviewed for epidemiological data.

**RESULTS:**

Forty-three cases of sporotrichosis were diagnosed through the period. The sample comprised predominantly young male adults and rural workers. The most common disease type was lymphocutaneous (51%), followed by fixed cutaneous form (32.5%). The predominant location was the upper limbs (70%), followed by the lower limbs (16%). A significant association was observed between the lymphocutaneous form and upper limb location and between the fixed cutaneous form and lower limb location (p = 0.019). Potassium iodine and itraconazole were the most common treatments.

**CONCLUSIONS:**

This study will help update the epidemiological situation of sporotrichosis in the central region of the state of Rio Grande do Sul, Brazil, over the last decade.

## INTRODUCTION

Sporotrichosis is a subcutaneous mycosis with a worldwide distribution that is currently notable for areas of especially high endemicity in Latin America. The disease shows a close correlation with environmental humidity, as high levels favor fungal propagation^1^. The infection usually reaches the deeper skin layers through implantation of the fungus caused by minor local trauma or animal bites and scratches and more commonly affects outdoor workers^2^ ^,^ ^3^. The lymphocutaneous form of the disease has the highest incidence, followed by the fixed cutaneous form^4^ ^-^ ^6^. The disseminated cutaneous form is rare and usually affects immunosuppressed individuals^2^ ^,^ ^5^.

Previous epidemiological studies revealed that sporotrichosis is rare, even in the Brazilian territory, where its prevalence is higher than that in other areas. A study in Vitória, Espírito Santo showed only 18 cases of sporotrichosis between 2005 and 2012^7^. At the Evandro Chagas Institute in Rio de Janeiro, 759 cases of sporotrichosis were diagnosed between 1998 and 2004^8^. A study of cats and dogs in the city of Pelotas, Rio Grande do Sul revealed 103 cases of sporotrichosis between 2000 and 2010^4^. In neighboring countries (e.g., Venezuela), studies reported a prevalence of 133 cases between 1963 and 2009^9^.

This study aimed to characterize the prevalence and characteristics of patients with sporotrichosis diagnosed at a tertiary university hospital over a 10-year period. 

## METHODS

This descriptive study (case series) was performed at Santa Maria University Hospital, a tertiary university public hospital located in the south of the Brazilian territory, a reference for approximately 550,000 people. From January 2006 to December 2015 we selected all cases with sporotrichosis diagnosed by microscopic examination or fungal isolation. Clinical records were reviewed for patients’ demographic information, clinical characteristics of the disease, and medical treatments. 

Data were processed and analyzed using SPSS version 16.0 software (SPSS Inc, Chicago IL, USA). For continuous variables, the Shapiro-Wilk test was used to determine the normality of the distribution. Parametric data were analyzed by Student’s t-test, while non-parametric data were analyzed by the Chi-square test. The level of statistical significance adopted was 0.05. The study adhered to the ethical criteria of the Declaration of Helsinki and was approved by the local research ethics committee.

## RESULTS

### Epidemiological Profiles

A total of 43 patients [31 (72%) men; mean age, 40.2 ± 16 years; median age, 43 years (interquartile range, 28-55 years)] with sporotrichosis were identified. The patients’ clinical characteristics are described in Table 1.

**TABLE 1:** Epidemiological profile of patients with sporotrichosis diagnosed in 2006-2015 at Santa Maria University Hospital.

Epidemiologic profile	N = 43 (100%)
Age¥	43 (28-55)
**Sex**	
Male	31 (72%)
Female	7 (16%)
HTN	8 (18,5%)
DM	2 (4,50%)
**Smoking status**	
Active	6 (14%)
Ex-smoker	3 (7%)
Asthma/COPD	1 (2,50%)
CKD	1 (2,50%)
Liver disease	2 (4,50%)
Alcoholism	3 (7%)

¥Median and interquartile range (non-parametric distribution). **CKD**: chronic kidney disease; **COPD**: chronic obstructive pulmonary disease; **DM:** diabetes mellitus; **HTN:** hypertension.

The occupations found were distributed as follows: 17 (39.5%) farmers, 3 (7%) construction workers, 2 (4.5%) students, 2 (4.5%) domestic workers, 1 (2.5%) driver, 1 (2.5%) teacher, and 1 (2.5%) laboratory worker. In 16 cases (37%), occupations were not recorded in the medical records.

### Characteristics of Lesions and Correlations

Regarding lesion distribution throughout the body, we found the following pattern: 30 cases (70%) in the upper limbs, 7 (16%) in the lower limbs, and 3 (7%) in other body locations (back, trunk, and disseminated). The medical records lacked lesion location descriptions in 3 cases. Regarding sporotrichosis classification, 14 cases (32.5%) were classified as fixed cutaneous, 22 (51%) as lymphocutaneous, and 1 (2.5%) as disseminated cutaneous. Six cases (14%) were not properly classified. 

In the present study, there was no association between the lesion location and professional occupation or between lesion location and sex (Fisher’s exact test, p > 0.05). However, there was a significant association between disease classification and lesion location. Among the 22 patients with the lymphocutaneous form, 20 (91%) presented with lesions in the upper limbs. On the other hand, patients with the fixed cutaneous form (14 cases) revealed a more diffuse pattern of distribution: 7 (50%) had lesions predominantly in the upper limbs (79 [50%]), 5 (36%) had lesions in the lower limbs, and 2 (14%) had lesions in other locations (Fisher’s exact test, p = 0.019). 

The results are specified in Table 2.

**TABLE 2:** Characteristics of sporotrichosis lesions**.**

Location of lesions [N = 43 (100%)]					
Upper limbs	Lower limbs	Other locations			Non-registered
30 (70%)	7 (16%)	3 (7%)			3 (7%)
		Trunk	Torso	Disseminated	
		1 (2.30%)	1 (2.30%)	1 (2.30%)	
**Classification sporotrichosis [N = 43 (100%)]**					
**Fixed cutaneous**	**Lymphocutaneous**	**Disseminated cutaneous**	**Extra-cutaneous**	**Non-registered**	
14 (32.50%)	22 (51%)	1 (2.50%)	0 (0%)	3 (7%)	
**Correlation between lesion location and sporotrichosis form**					
			**Classification of sporotrichosis**		
			**Fixed cutaneous**	**Lymphocutaneous**	
			**14 (100%)**	**22 (100%)**	
	Lesion location	Upper limbs	7 (50%)	20 (91%)*	
		Lower limbs	5 (36%)*	2 (9%)	
		Other	2 (14%)	0 (0%)	

The * indicates a significant association between the lymphocutaneous form and an upper limb location as well as fixed cutaneous form and lower limb location (Fisher’s exact test, p = 0.019).

### Treatment

There was no clinical protocol to guide patient treatment at our institution. Potassium iodide was the preferred medication in 24 cases (56%). This medication showed reasonable cure rates; while 16 patients (66.6%) were cured, 5 (20.8%) needed new treatments, and 3 (12.5%) missed outpatient follow-up. All 5 therapeutic failures were switched to itraconazole, and one patiens ended up being cured only with surgical excision of the lesion.

Itraconazole was the first choice in 11 (25.5%) cases. With this treatment option, cure was achieved in 6 cases (54.5%). Seven cases (16%) required multiple treatments modalities. 

The treatment time was variable, with a median of 4.8 months (interquartile range [IQR], 2.1 - 8.5). Some unusual cases had a much longer treatment time (e.g., 47 months of treatment). The median treatment with potassium iodide (5.7 months; IQR, 2.3-9.5) was not significantly different from that of itraconazole (3.3 months; IQR, 1.9-4.7; p = 0.1).

Likewise, treatment time in men (4.6 months; IQR, 2.0-7.0) was not significantly different from that in women (9.2 months, IQR, 2.6-18.0; p = 0.26).

## DISCUSSION

The present study describes a series of 43 sporotrichosis cases diagnosed in a tertiary university hospital located in the central region of Rio Grande do Sul, Brazil over the last 10 years. The Santa Maria University Hospital is a teaching hospital that serves as a reference for cutaneous infectious diseases and covers a population of approximately 541,247 inhabitants.

Previous studies in the same geographical region have shown a progressively lower number of cases over the last few decades. Londero et al. showed 117 cases between 1957 and 1967, a number that decreased to 31 between 1988 and 1997. Considering the epidemiological limitations of our study, which has no power to evaluate disease incidence, the tendency of increasing cases in the last decade in the central region of Rio Grande do Sul, Brazil, is plausible. To confirm this hypothesis, prospective studies are needed^10^ ^,^ ^11^.

The present study also determined the main characteristics of individuals with sporotrichosis. Like other studies, we observed a pattern of involvement in young male adults and rural workers^9^ ^,^ ^10^ ^,^ ^12^ ^-^ ^16^. This predisposition may be related to our population, who mostly inhabited rural areas. In these areas, fungal transmission predominately occurs through skin trauma such as animal bites (cats, rodents) and objects contaminated with *Sporothrix* spp*.* (spines, wood chips, or contaminated organic material). 

Regarding lesion location, our results agree with most data in the literature showing that lesions are more frequently located in the upper limbs^9^ ^,^ ^10^ ^,^ ^12^ ^-^ ^14^. These data support the hypothesis that lesions start from local trauma after the manipulation of objects (farmers) or animal scratches or bites^8^ ^,^ ^17^. Interestingly, our study demonstrated a significant association between lymphocutaneous form and location in the upper limbs, confirming data found in the literature that suggest dissemination through the lymphatic pathway after the initial injury^11^ ^,^ ^14^ ^,^ ^18^ ^,^ ^19^. In the same way, our study demonstrated an association between the fixed cutaneous form and the lower limb location. Few published studies have shown this association, allowing only speculative hypotheses^11^ ^,^ ^18^ ^,^ ^19^. 

We observed that most patients (56%) received initial treatment with potassium iodide. Itraconazole was usually the second choice. Most medical centers adopt the same treatment options described in our study, with similar efficacy. Both medications are recommended by current guidelines as first choices for the treatment of the fixed cutaneous and lymphocutaneous forms of sporotrichosis. At our institution, we prefer to use potassium iodide as a first-line treatment, mainly due to cost effectiveness^20^. We also observed wide variation in treatment period (median 4.8 months; IQR, 2.1-8.5). Three patients required prolonged treatment (up to 47 months), and treatment adherence was the main concern for low cure rates. There was no significant intergroup difference in median treatment time (5.7 months for potassium iodide vs. 3.3 months for itraconazole; p > 0.05)^13^ ^,^ ^14^ ^,^ ^21^.

Although these findings are important for the delineation of the epidemiological, clinical, and therapeutic profiles of sporotrichosis in the central region of Rio Grande do Sul in the last decade, our study has several limitations. We initially highlighted the methodological limitations of a cross-sectional study. The data collection through medical record review led to some incomplete (or even incorrect) data. Despite our hospital being a reference facility for region-wide sporotrichosis treatment, cases may have been missing, including patients who received treatment at other institutions or did not receive treatment at all. 

Despite these limitations, the study helps update the epidemiological status of sporotrichosis in the central region of Rio Grande do Sul, Brazil.
